# Ultrasonic Cutting of Frozen Semen Straws to Optimize the Use of Spermatozoa for In Vitro Fertilization

**DOI:** 10.3390/ani10112152

**Published:** 2020-11-19

**Authors:** Sung Woo Kim, Jae-Yeong Lee, Bongki Kim, Chan-Lan Kim, In-Sul Hwang, Yeoung-Gyu Ko, Sung-Soo Lee

**Affiliations:** 1 Animal Genetic Resource Research Center, National Institute of Animal Science, RDA, Hamyang 50000, Korea; jay0i@korea.kr (J.-Y.L.); vetchan@korea.kr (C.-L.K.); kog4556@korea.kr (Y.-G.K.); lee6470@korea.kr (S.-S.L.); 2 Department of Animal Resources Science, Kongju National University, Yesan 32439, Korea; bkkim@kongju.ac.kr; 3 Animal Biotechnology Division, National Institute of Animal Science, RDA, Wanju 55365, Korea; insuri2642@korea.kr

**Keywords:** sperm viability, embryo production, fertilization, blastocyst

## Abstract

**Simple Summary:**

The use of frozen semen is essential for the success of in vitro fertilization in the bovine embryo transfer industry. Therefore, we aimed to maximize the use of a single frozen semen straw by employing a cutting protocol. To the best of our knowledge, the current study is the first to apply ultrasonic cutting to frozen semen. The mechanical damage of spermatozoa was studied in frozen bovine semen by assaying sperm motility, acrosome abnormalities, and developmental competence. These findings may help to optimize the utilization of frozen semen for in vitro fertilization in the bovine industry.

**Abstract:**

The objective of the present study was to establish conditions for using technology that can potentially enhance the efficiency of bovine embryos derived from in vitro fertilization (IVF) with frozen semen. Frozen semen from selected bulls can be stored indefinitely in liquid nitrogen as genetic resources; however, these resources are considered consumable because they cannot be regenerated. Therefore, to optimize the utilization of frozen semen, as many oocytes as possible should be fertilized with one straw. However, a sufficient number of prepared oocytes might not be available for one experiment, which can limit the use of the total spermatozoa population. Thus, an economical method for producing embryos needs to be established by optimizing technology for transplantable embryos. In this study, the utilization of frozen semen was increased by dividing the straw with an ultrasonic cutter. The post-thaw survival rate of uncut straws from Korean Proven Bulls did not differ from that of half cuttings. When ultrasonic cutting was applied to frozen semen, spermatozoa could be prepared for IVF trials at least four times, and blastocysts were produced. Therefore, cutting frozen semen with an ultrasonic cutter represents a potentially useful tool to expand genetic resources from excellent breeding stocks. This approach could also be valuable in the field of IVF of endangered species or rare breeds for their preservation, as well as in ovum pick-up (OPU) techniques.

## 1. Introduction

In vitro bovine embryo production using in vitro fertilization (IVF) procedures was first introduced for the embryo transfer of livestock [1,2,3]. Much attention has been drawn to the mass production of transplantable blastocysts to preserve genetic resources [4,5,6]. For this purpose, spermatozoa with optimal activity and motility are an essential and indispensable element of IVF using frozen semen [7,8]. However, lab workers and farmers face difficulties in purchasing frozen semen with good genetic backgrounds because of its high demand. Moreover, to prevent the inbreeding of *Hanwoo*, the government of South Koreahas placed limitations on the specific supply of semen from the highly preferred Korean Proven Bull Number (KPN) family.

Since the IVF technique was developed with immature oocytes from slaughterhouse-derived or in vivo ovaries, IVF technicians have become aware of the importance of sperm physiology to produce healthy embryos with suitable culture systems [1,3,9]. Recently, the ovum pick-up (OPU) technique was developed for multiple oocyte recovery from a single cow with 8~15 oocytes per collection, so animal farmers have focused on genetic improvements for subsequent transferable blastocysts [5,10,11,12]. The estimated number of spermatozoa from a single frozen semen sample needed for IVF or artificial insemination (AI) can differ. Approximately 15 × 10^6^ to 20 × 10^6^ active spermatozoa are required for one bovine AI [13,14,15]. However, the number of spermatozoa needed for IVF trials has been reported to be approximately 3 × 10^3^ to 5 × 10^3^ active spermatozoa per oocyte [14,15]. These requirements mean that a significant number of spermatozoa are not used in the IVF procedure, and unused sperm is discarded as laboratory waste. Therefore, the ability to leverage multiple uses from one straw of frozen semen could increase the productivity of genetically confirmed spermatozoa, and such a process with high efficiency could lead to a new protocol for IVF researchers.

The ultrasonic cutter, which has a blade that vibrates 20,000–40,000 times per minute, has been developed to gently and accurately cut high-density materials, such as plastic assembly toys [16,17,18]. In this study, an ultrasonic cutter was tested to obtain multiple uses of frozen semen. It was hypothesized that the characteristics of this instrument would allow a hard frozen straw to be cut quickly and cleanly. In general, materials submerged in liquid nitrogen (LN_2_) have increased hardness, show very rigid physical properties, and are not cut easily by scissors [19]. When straw-cutting scissors are used to cut semen straws while they are in the frozen state, cracks are formed on the straw’s plastic surface, which leads to semen loss during thawing. Therefore, the present study was designed to offer a new method to produce cuttings with an ultrasonic cutter without thawing; in this approach, the frozen semen straws can be maintained in preservation conditions below the surface of LN_2_. To the best of our knowledge, the ultrasonic cutting technique has not been used for cutting frozen semen, and data on the sperm properties from divided frozen semen could not be found. Thus, the present study was conducted to present accurate data for this field and demonstrate the method’s feasibility. In this study, the level of damage to spermatozoa from cuttings was investigated by monitoring their motility and activity. Moreover, the production of IVF embryos using the spermatozoa was investigated to confirm the performance of fertilizing activity from frozen semen cuttings.

## 2. Materials and Methods

### 2.1. Animals and Ethics Statement

Ovaries from the Korean Hanwoo cow were obtained from a local slaughterhouse (Iksan-si, Jeollabuk-do province). All procedures described were reviewed and approved by the Institutional Animal Care and Use Committee at the National Institute of Animal Science (Approval No. NIAS 2018–406).

### 2.2. Frozen Bovine Semen and Semen Motility Assay

Commercially available frozen semen from KPN was chosen because of the certified grade of sperm quality. The frozen semen was thawed at 37.5 °C for 45 s, and the sperm was transferred into a 1.5 mL tube on a digital warming block (EppendorfThermoMixer^TM^, Hamburg, Germany) without shaking. After placing 10 µL of thawed spermatozoa samples on the Makler counting chamber (Sefi-Medical Instruments, Haifa, Israel), a comparative analysis of the motility and activity was performed using a microscope with a computer-assisted sperm analyzer (CASA) from the Proiser R+D corporation in Spain.

### 2.3. Bovine Semen Collection, Dilution, and Freezing

Ultrasonic cutting experiments were performed using frozen semen from three Jersey breeding bulls. The semen samples were collected using an electro stimulator (Pulsator IV, Lane Manufacturing Inc., Denver, CO, USA) and cryopreserved. The collected semen samples were transported to the laboratory within 10 min and diluted (1:1) by the dropwise method using Triladyl^TM^-egg yolk diluent. The diluted semen samples were left at room temperature for 10 min and then diluted again to adjust the final spermatozoa concentration to 80 × 10^6^/mL. A double cooling system was prepared by adding room-temperature water to a ten-fold larger volume of semen in a plastic beaker. The semen-containing vessel was cooled for 2–3 h using slurry ice. After cooling it to 5 °C, the semen was packed into 0.5 mL straws by an automated filling and sealing machine (MPP Uno, Minitube, Tiefenbach, Germany) in a semen processing cabinet (FHK, Tokyo, Japan). The packaged semen samples were frozen by exposure to LN_2_ by first placing them 5 cm above the tank for 10 min and then submerging them.

### 2.4. Ultrasonic Cutting and Thawing of the Frozen Semen Straw

**Figure 1:**
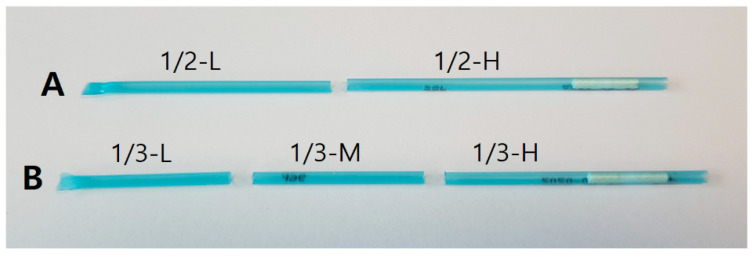
Images of the samples after applying the cutting treatment to hollow 0.5 mL straws. The hollow straw (**A**) is divided into two cuttings, each with one opening;1/2-H is a cutting from the top portion with a cotton seal, and 1/2-L is a cutting from the lower portion with a sealed end. The hollow straw (**B**) is cut twice, producingthree cuttings; 1/3-M is the middle portion of the straw with two openings.

**Figure 2:**
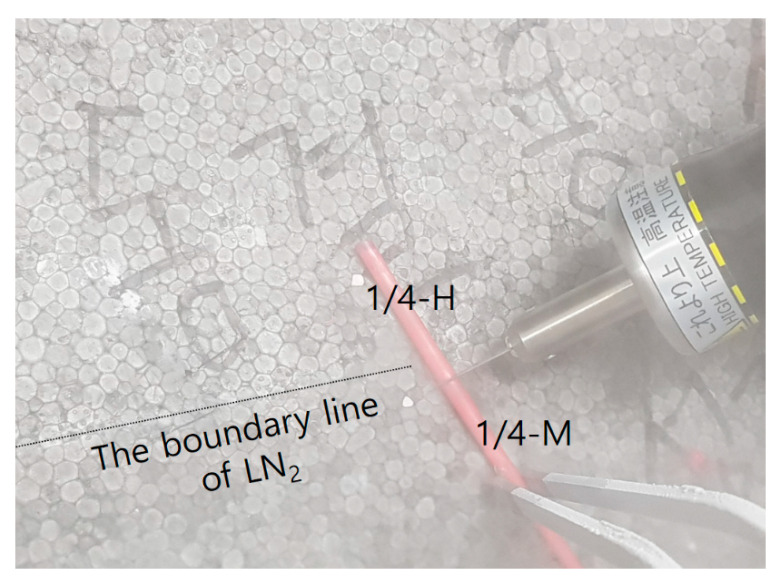
An image of the ultrasonic cutter on the frozen semen straw. The bottom of the tilted Styrofoam box was used to expose the cutting area of frozen semen to the surface of LN2.

The frozen semen straw fragments were obtained by using an ultrasonic cutter (Honda Electronics, ZO-80, Toyohashi, Japan). First, the intact straw was placed into a tilted Styrofoam box filled with LN_2_ that covered half of the tilted bottom. As shown in Figure 1 and Figure 2, the straws were cut smoothly with the ultrasonic cutter for 2–3 s at the surface boundary of LN_2_, in which the straws were partially submerged. The 1/2 or 1/3 cuttings of the whole straw remained in LN_2_ and were returned to the straw goblet for preservation. The cuttings of frozen semen with two openings were placed into a thin and small plastic bag and submerged in 37.5 °C water for 45 s. The openings of the cutting were blocked and kept horizontal by hand to prevent the spillage of semen. The 1/4 cutting of frozen semen was placed directly into a 15 mL conical tube with 13 mL of S-BO medium, which was warmed at 38.5 °C in the incubator. The tube was inverted 6–8 times to increase the thawing speed of semen samples. The remaining empty straw was removed with sterilized forceps before centrifugation. To fertilize the control oocytes with sperm from uncut frozen straws, the semen was mixed with calcium-/magnesium-free phosphate-buffered saline (CMF-PBS) in a ratio of 1:9 by volume and centrifuged for 10 min at 300 g to remove the diluent component.

### 2.5. Acrosome Analysis by CBB Staining

**Figure 3:**
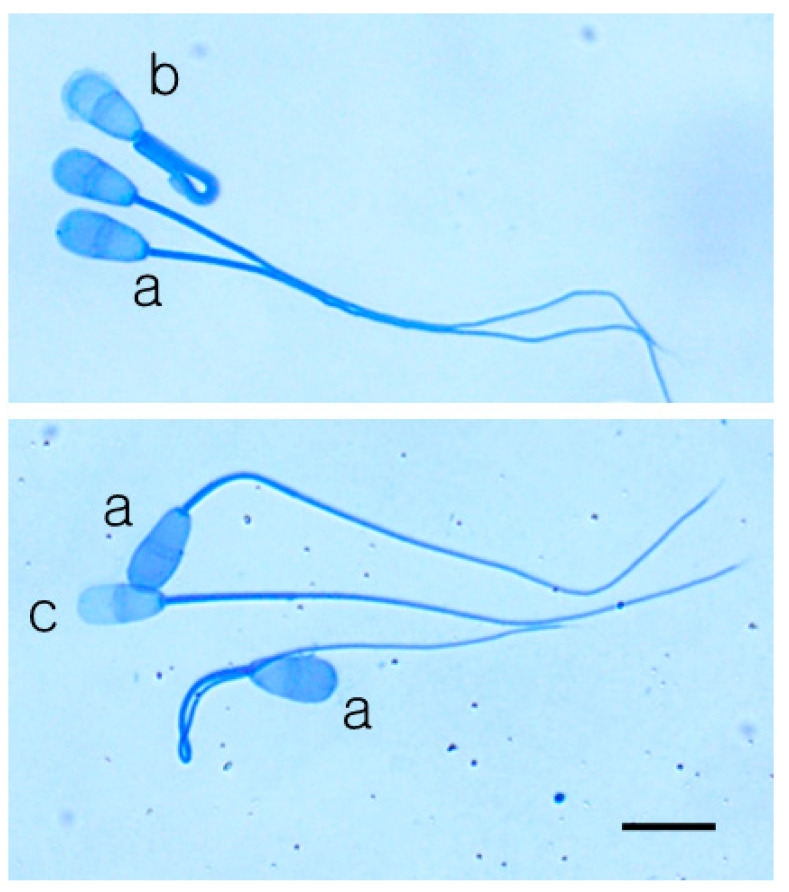
A bovine spermatozoa image stained with a mixed solution of 0.1% coomassie brilliant blue (CBB) R-250 and 0.1% CBB G-250. Spermatozoa with intact acrosomes (**a**) have a bright blue staining pattern over the entire acrosome, whereas damaged spermatozoa have swollen outer membranes (**b**) or no staining pattern over the acrosome (**c**). The black bar is 10 μm.

The sperm pellets were suspended by gentle tapping. A 5 µL drop of spermatozoa was placed on a glass slide and smeared, dried, and fixed by submersion in PBS containing 3.7% paraformaldehyde (PF-PBS) for 2 min. Coomassie brilliant blue (CBB) stains were used to analyze the acrosome status. The CBB stock was prepared by dissolving 0.3 g of CBB staining reagent in 45 mL of methanol, and a diluent for CBB stock was prepared by mixing 10 mL of acetic acid with 45 mL of double-distilled water. The staining solution was prepared by adding the diluent to CBB stocks, and the final concentration of CBB reagents was 1% CBB R-250 and 1% CBB G-250. A fixed sperm smear was submerged in the staining solution for 2 min and transferred into distilled water for 3–5 s. After drying the stained smears on a slide warmer at 37 °C for 2–3 min, the slide was observed with a microscope (Olympus IX-71, Tokyo, Japan). As shown in Figure 3, the acrosome reaction status was analyzed by counting the number of spermatozoa with intact acrosomes, reacting acrosomes, and reacted acrosomes. The total number of counted sperm was over 200 per examination in each experiment.

### 2.6. In Vitro Fertilization of Bovine Oocytes with Sperm from the Ultrasonic Cuttings of Frozen Semen

Cumulus–oocyte complexes (COCs) were collected by aspiration from 3–6 mm follicles of slaughterhouse-derived ovaries using a 10 mL disposable syringe with an 18-gauge needle. The pooled COCs were washed three times using tissue culture medium(TCM) 199 culture medium containing 5% FBS to remove the follicular fluid and cells that had detached from COCs. The maturation medium was prepared with TCM 199 culture medium containing 10% FBS, FSH (20 µg/mL), LH (10 µg/mL), and estradiol (1 µg/mL) and incubated overnight in the previous day’s oocyte culture. The COCs were cultured for 20–22 h and subsequently fertilized by spermatozoa from the frozen semen straw. As a fertilization medium, Bracket and Oliphant(BO) culture medium was prepared for sperm treatment and fertilization drops for IVF. The fertilization BO drops (F-BO) were prepared by adding 6 mg/mL fatty acid-free bovine serum albumin (FAF-BSA). Drops of culture (25 µL) covered with mineral oil were incubated at 39.5 °C overnight. The semen was transferred to 15 mL centrifuge tubes with S-BO and centrifuged for 5 min at 250 g to remove the diluent of egg yolk and glycerol. To enhance sperm activity and induce capacitation, sperm dilution BO (S-BO) was prepared by adding 0.45 mg/mL theophylline. Approximately 20 COCs were transferred to F-BO, and 25 µL of S-BO containing spermatozoa at a concentration of 4 × 10^6^ to 7 × 10^6^/mL was co-cultured for IVF for 8–16 h.

### 2.7. Statistical Analysis

The rates of motility and damage to sperm cells were analyzed using a one-way analysis of variance (ANOVA) test, and the significance between the means of analyzed data was evaluated using Duncan’s multiple range test.

## 3. Results

### 3.1. Effects of Ultrasonic Cutting on Spermatozoa Motility

The motilities of KPN spermatozoa obtained from frozen cuttings were compared with those of the control semen, which was obtained from uncut frozen straws. As shown in Figure 2, the hollow straw samples were cut into 1/2 and 1/3 pieces; 1/2-L indicates the lower portion of the straw, and 1/2-H is the top portion, which resides in a goblet clipped to a metal cane in the preservation chamber of the LN_2_ tank. As shown in Table 1, the percentage of motile spermatozoa from 1/2-Ldid not differ from that of uncut frozen spermatozoa (control); however, the motility differed between 1/2-H and the control. The motility of spermatozoa from the lower portion of the frozen semen sample cut by 1/3 was 88.4% and had no significant differences (*p* > 0.05) from the control. However, the motility of spermatozoa from 1/3-H and 1/3-M significantly differed (*p* < 0.05) from that of the control. The percentage of hyperactive spermatozoa differed between 1/2-L, 1/2-H, and the control semen (*p* < 0.05). When the straw was cut by 1/3, the motilities of spermatozoa differed between 1/3-M, 1/3-H, and 1/3-L. There were also differences in hyperactive sperm rates between 1/3-M, 1/3-H, and 1/3-L. The same tendency of hyperactive sperm rates was also observed in the 1/2 cuttings, but those from the lower cutting differed as compared with the control. The slow motility was not significantly different between the 1/2 cuttings and the control. However, the slow motility of 1/3-M and 1/3-L differed from that of the control. Interestingly, spermatozoa concentrations and motility followed the same trend. The 1/2-H and 1/3-H cuttings contained fewer spermatozoa than 1/2-L, 1/3-L, and the control.

### 3.2. Effects of Ultrasonic Cutting on Acrosome Integrity

The acrosome reactions of spermatozoa in the samples were analyzed by CBB differential staining techniques. The acrosome reactions of spermatozoa from KPN frozen semen straws that were ultrasonically cut into 1/2 and 1/3 were compared with those of the control. As shown in Table 2, there were no significant differences in the rates of spermatozoa with intact or reacting acrosomes between the control, 1/2-L, and 1/2-H. However, the rates of reacted acrosomes in the sperm of 1/3-H and 1/3-M were significantly different from those of the control and 1/3-L (*p* < 0.05).

### 3.3. IVF Using the Spermatozoa from 1/4 Cuttings of Frozen Semen

**Figure 4:**
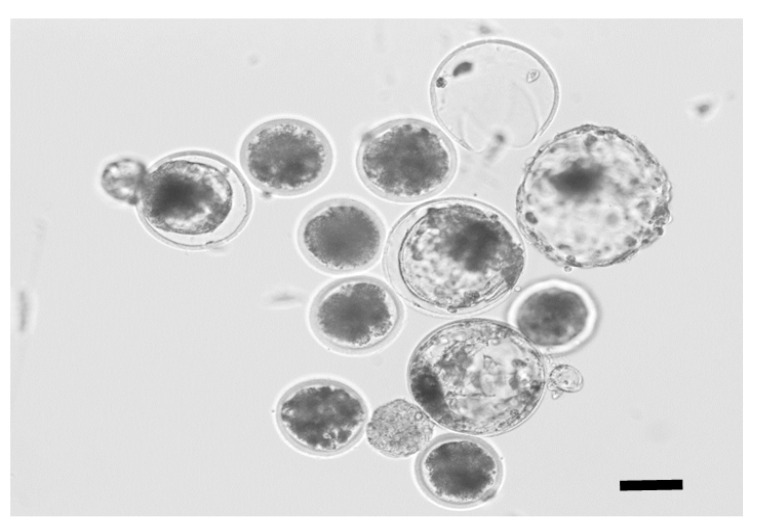
An image of in vitro fertilization (IVF)-derived blastocysts using spermatozoa from the 1/4-L cutting region of the semen straw. The black bar is 100 μm.

To test the developmental competence of spermatozoa from frozen semen cuttings, IVF was conducted using in vitro matured oocytes, which were collected from slaughterhouse-derived ovaries. As shown in Table 3, the developmental competence of the spermatozoa from the 1/4-H and 1/4-L cuttings of Jersey frozen semen was compared with that of the uncut control semen. The rate of blastocyst production onthe seventh day of culture did not differ between the control and 1/4 cuttings. One hatched and two hatching blastocysts from the sperm of 1/4-L cuttings are shown in Figure 4.

## 4. Discussion

Polyvinyl plastics, which are used as the soft material of semen straws, are characterized by their increased rigidity and strength at ultra-low temperatures (for example, the temperature of LN_2_), which are also needed for the cryopreservation of frozen semen. The blade of an ultrasonic cutter vibrates 20,000~40,000 times per second (20~40 kHz), and the friction force from the blade quickly raises the temperature between the blade surface and the object being cut [18]. These properties have been used to cut hard objects such as plastic models and are employed in devices such as butter cutters; as a result of these effects, the cutting force applied to the materials is reduced [16,17]. However, to the best of our knowledge, the application of an ultrasonic cutter has not been studied in cryobiology to cut frozen semen straws.

The present study was based on the potential use of an ultrasonic cutter to cut frozen semen while minimizing damage and preventing cracks that cause spillage during the thawing process. When the cutter is applied to frozen semen, the effects on the spermatozoa in the straws can include lowered motility and acrosome damage. The fertility of damaged spermatozoa in IVF trials may also be compromised. In the present study, the results obtained using semen cuttings from nationally managed KPN Hanwoo and Jersey breeds demonstrate the capability of ultrasonic cutting. Moreover, the fertilizing ability of frozen–thawed spermatozoa derived from the cuttings was evaluated by assessing the developmental competence of blastocysts resulting from IVF procedures. As shown in Figure 2, the frozen semen straw to be cut was only exposed at the surface boundary of LN_2_ to reduce the thawing effect from the heat that was generated by friction between the blade and straw. A Styrofoam box with a tilted bottom was used to keep straws submerged in LN_2_, which was expected to ensure effective preservation. This assumption can be validated by measuring the motility of spermatozoa from cuttings.

Interestingly, the motility of frozen–thawed semen from the lower positions of straws cut by 1/2 and 1/3 was higher than that from other regions. These results suggest that the motility of sperm from the top region of the goblet may be decreased by temperature fluctuations, which may be caused by LN_2_ evaporation. Therefore, the sperm in the lower region of the goblet may have comparatively high motility. Some spermatozoa from the middle portion of the cut straw appeared to be damaged, although the difference was not significant. During the thawing process, the two openings of the middle cuttings can result in the spillage of semen. However, these damages and losses did not affect the motility of the sperm population for the IVF procedure. It was predicted that only a small portion of sperm in the cut area would be lost during the thawing process and that cutting would not affect the characteristics of the overall sperm population. Moreover, the lower region of the goblet was the most protected area by LN_2_ because it remained submerged during the transportation of frozen semen. The sperm from the lower region in the goblet had the best motility. The investigation of acrosome damage also verified that sperm from the top portion of the frozen semen straw generally had a greater percentage of damage, as evidenced by acrosome reactions that were in progress or completed. Therefore, the ultrasonic vibration force does slightly damage the spermatozoa from the cutting area, but it does not appear to have a significant effect on the total population of spermatozoa or their availability. As described earlier, the bovine IVF protocol only uses a portion of thawed semen, so only selected spermatozoa are used in fertilization. The results of this study suggest that the proposed method may also be applicable to the bovine OPU technique. Using frozen semen in high demand from bulls with good genetic backgrounds, an elite breeding stock can be produced with the repeated use of a single frozen semen straw. Since this method allows IVF researchers to use one frozen semen sample in four trials or more, the semen cost can be lowered.

## 5. Conclusions

Recently, in Korea, an increasing number of farms have attempted to transfer OPU-derived blastocysts for breeding as a method for improving the genetic ability of their elite females. However, it is not easy to secure or purchase frozen semen from desirable males for various reasons. By applying the ultrasonic cutting technique proposed in the present study, such problems can be easily resolved with the use of high-quality frozen semen. Therefore, it is concluded that the ultrasonic cutting technique can be applied to frozen semen for IVF and may be used as an efficient method of semen treatment to build an elite breeding stock.

## Figures and Tables

**Table 1:** The effects of ultrasonic cutting on the sperm motility and activity of Korean Proven Bull Number (KPN) spermatozoa.

Cutting Treatment	Concentration (×10^6^/mL)	% of Spermatozoa		
		Motile	Hyperactive	Slow
Control	115.1 ± 2.0	84.9 ± 1.6	68.5 ± 3.1	16.5 ± 1.9
1/2-H	48.7 ± 7.9 *	77.8 ± 0.6 *	61.1 ± 3.6 *	16.8 ± 4.2
1/2-L	119.0 ± 24.5	88.2 ± 1.5	77.4 ± 4.2 **	10.8 ± 5.1
1/3-H	94.5 ± 4.6 *	76.8 ± 2.5 *	64.7 ± 5.9 *	12.1 ± 4.7
1/3-M	86.3 ± 22.9 *	72.7 ± 1.9 *	61.7 ± 0.7 *	11.0 ± 1.6 *
1/3-L	143.9 ± 25.0	88.4 ± 1.3	78.4 ± 1.4 **	10.0 ± 2.6 *

H: cuttings from the top portion with a cotton sealing component; L: cuttings from the lower portion without a cotton sealing component; M: cuttings from the middle portion with two openings. Mean values that are statistically significant different between treatments are indicated by different superscripts * and ** (*p* < 0.05). Data are expressed as the mean ± SD from three independent experiments.

**Table 2:** The effects of ultrasonic cuttings on the acrosome integrity of KPN spermatozoa.

Cutting Treatment	% of Spermatozoa		
	Intact	Reacting	Reacted
Control	75.4 ± 3.0	20.9 ± 2.5	3.8 ± 0.6
1/2-H	70.6 ± 13.8	19.6 ± 8.6	9.8 ± 5.3
1/2-L	77.6 ± 4.4	17.1 ± 2.8	5.3 ± 2.0
1/3-H	67.5 ± 7.6	21.8 ± 7.4	10.7 ± 0.4 *
1/3-M	71.6 ± 2.4	14.5 ± 4.5	13.9 ± 2.2 *
1/3-L	78.2 ± 4.5	17.8 ± 2.8	4.0 ± 1.8

H: cuttings from the top portion with a cotton sealing component; L: cuttings from the lower portion without a cotton sealing component; M: cuttings from the middle portion with two openings. Mean values that have statistically significant differences between treatments are indicated by different superscripts * (*p* < 0.05). Data are expressed as the mean ± SD from three independent experiments.

**Table 3:** The effects of ultrasonic cutting treatment on the developmental competence of spermatozoa.

Cutting Treatment	n	% of Spermatozoa		
		Cleaved	Morula	Blastocyst
Control	66	54.5 ± 11.2	30.2 ± 6.3	22.6 ± 3.5
1/4-H	71	60.1 ± 13.1	28.2 ± 15.6	20.0 ± 6.3
1/4-L	69	53.3 ± 20.7	36.8 ± 10.0	26.2 ± 2.3

H: cuttings from the top portion with a cotton sealing component; L: cuttings from the lower portion without a cotton sealing component. Data are expressed as the mean ± SD from three independent experiments.
